# Dietary B-complex vitamins and sleep quality in relation to cognitive impairment among older adults: non-linear associations and evidence of additive interaction

**DOI:** 10.3389/fnut.2025.1694999

**Published:** 2025-12-19

**Authors:** Meihui Zhang, Yuxuan Wang, Yinghuan Zhang, Quyige Gao, Fan Hu, Ying Wang, Chunhai Shao, Yong Cai

**Affiliations:** 1 School of Public Health, Shanghai Jiao Tong University, Shanghai, China; 2 Public Health Research Center, Tongren Hospital, Shanghai Jiao Tong University School of Medicine, Shanghai, China; 3 Department of Clinical Nutrition, Huashan Hospital, Fudan University, Shanghai, China; 4 Department of Clinical Nutrition, Shanghai Fifth People's Hospital, Fudan University, Shanghai, China; 5 Center of Community Healthcare, China Hospital Development Institute, Shanghai Jiao Tong University, Shanghai, China

**Keywords:** cognitive aging, B-complex vitamins, sleep quality, nutritional epidemiology, prevention

## Abstract

**Background:**

Global dementia cases are projected to reach 152.8 million by 2050, with China accounting for approximately 25% of cases. While B-complex vitamins and sleep quality are established modifiable determinants of cognitive health, their potential synergistic effects on cognitive function remain unexplored, particularly in rapidly aging populations.

**Methods:**

In this population-based cross-sectional study, we analyzed baseline data from 8,806 adults aged ≥65 years in the Shanghai Aging and Retirement Evaluation Survey (January-May 2024). Dietary B-complex vitamins intake was assessed using a validated Food Frequency Questionnaire. Sleep quality was evaluated using the Pittsburgh Sleep Quality Index (PSQI). Cognitive function was measured using the Mini-Mental State Examination, with scores ≤ 24 indicating cognitive impairment. We examined non-linear relationships using restricted cubic splines analysis and assessed independent associations using multivariate logistic regression. Both multiplicative and additive interactions were evaluated using cross-product terms and three additive interaction indices.

**Findings:**

Among 8,806 participants, 677 (7.69%) had cognitive impairment. Higher intake of vitamin B6, B12, and folate showed L-shaped associations with reduced cognitive impairment risk, while PSQI scores demonstrated J-shaped relationships. Compared with lowest quartiles, participants in highest quartiles of vitamin B6, B12, and folate intake had lower odds of cognitive impairment (adjusted OR 0.33 [95% CI: 0.25–0.43], 0.43 [95% CI: 0.33–0.55], and 0.33 [95% CI: 0.25–0.43], respectively; all *p* < 0.001), while those with highest PSQI scores had increased odds (2.94 [95% CI: 2.34–3.69], *p* < 0.001). Significant additive interactions between low B-complex vitamins intake and poor sleep quality were observed (Relative Excess Risk Due to Interaction 0.97 [95% CI: 0.48–1.46]; Attributable Proportion 0.22 [95% CI: 0.10–0.35]; Synergy Index 1.41 [95% CI: 1.14–1.67], all *p* < 0.001), suggesting a synergistic association with cognitive impairment risk.

**Interpretation:**

Our findings reveal a synergistic association of adequate B-complex vitamins intake and good sleep quality with a lower prevalence of cognitive impairment in older adults, suggesting that integrated interventions targeting both nutritional and sleep factors might be more effective than single-domain approaches in preserving cognitive health during aging.

## 1 Introduction

Cognitive decline and dementia represent major challenges to healthy aging globally, with devastating impacts on individuals, families, and healthcare systems. The global burden of dementia is projected to nearly triple from 57.4 million cases in 2019 to 152.8 million by 2050 (1), with disproportionate increases anticipated in low- and middle-income countries. China faces an unprecedented challenge, accounting for approximately 25% of global cases and experiencing the world's most rapid population aging (2). With cognitive impairment now ranking as the fifth leading cause of mortality in China (3), identifying modifiable risk factors for early intervention has become a public health imperative, particularly given the limited efficacy of current therapeutic options.

Emerging evidence suggests that modifiable lifestyle factors, particularly nutritional status (4) and sleep patterns (5), may play crucial roles in cognitive aging. Among these, B-complex vitamins and sleep quality have emerged as promising targets for intervention, supported by both mechanistic insights and epidemiological evidence. However, their potential synergistic effects remain largely unexplored, particularly in Asian populations experiencing rapid nutritional and lifestyle transitions.

B-complex vitamins, especially vitamin B-6, vitamin B-12, and folate, act as methyl donors or essential coenzymes for 1-carbon metabolism (OCM), directly influencing DNA methylation and nucleotide synthesis, thus play a critical role in AD pathogenesis (6). In addition, deficiency in B-complex vitamins can induce elevated levels of plasma homocysteine (Hcy), which can cause central nervous system damage, cognitive impairment, dementia, and AD (7). Supporting this mechanistic link, a large-scale prospective cohort study from the UK Biobank demonstrated that higher dietary intake of B-complex vitamins was associated with a significantly reduced risk of AD, particularly in individuals with high genetic susceptibility (8). Moreover, a comprehensive systematic review which analyzed 95 studies found that B vitamins supplementation significantly slowed cognitive decline, particularly in people living without dementia receiving long-term intervention (>12 months) (9). These results underscored the need to evaluate B vitamins within holistic dietary patterns, particularly in aging populations undergoing nutritional transitions.

Sleep quality has emerged as a crucial determinant of cognitive health in aging populations, with its disruption increasingly recognized as a modifiable risk factor for cognitive decline. Global epidemiological data suggest a concerning trend in sleep disturbances among older adults, particularly in rapidly urbanizing societies. In China, a recent meta-analysis of 32 studies comprising 376,824 participants estimated the pooled prevalence of poor sleep quality in the general population at 19.0% (10), reflecting broader sociodemographic and lifestyle transitions. Multiple biological mechanisms link poor sleep quality to cognitive impairment. Sleep disruption impairs the glymphatic system's clearance of neurotoxic waste products, notably promoting amyloid-β peptide accumulation in brain regions critical for memory and executive function (11). Additionally, chronic sleep deficiency triggers neuroinflammatory cascades and disrupts synaptic plasticity (12), potentially accelerating neurodegenerative processes. Supporting these mechanistic insights, longitudinal studies have demonstrated that poor sleep quality predicts both lower baseline cognitive performance and accelerated cognitive decline. In a multi-ethnic cohort of older adults, poor sleep quality was independently associated with faster deterioration of executive function (13), while a nationwide study of Chinese adults revealed robust associations between sleep quality metrics and cognitive performance across multiple domains (14).

Despite extensive research on B-complex vitamins and sleep quality in cognitive aging, several critical knowledge gaps persist. First, evidence regarding their independent associations with cognitive function remains inconsistent (8, 13–17), potentially reflecting methodological heterogeneity in assessment tools, inadequate control for confounding factors, and population-specific variations in exposure patterns. Second, the potential synergistic effects between these factors have been largely overlooked, despite compelling biological evidence suggesting shared pathways involving homocysteine metabolism (7, 18), oxidative stress (19, 20), and neuroinflammatory regulation (21, 22). This oversight is particularly significant given that B-vitamins and sleep quality may interact through multiple mechanisms to influence cognitive outcomes. Third, while evidence predominantly stems from Western populations, data from Asian contexts remain limited, despite marked differences in dietary patterns, sleep behaviors, and genetic backgrounds. This gap is especially notable in China's rapidly aging population, where unique sociocultural factors influence both nutritional status and sleep patterns. Understanding these population-specific associations and potential interactions could inform more effective, culturally-tailored strategies for cognitive health preservation in aging societies.

To address these knowledge gaps, we investigated both independent and synergistic associations between B-complex vitamins intake and sleep quality with cognitive function in a large population-based cohort of older adults in Shanghai, China using data from the Shanghai Aging and Retirement Evaluation (SHARE) study. First, we provided crucial evidence from a Asian population, addressing the critical gap in Asian-specific data on these relationships. Second, we examined both multiplicative and additive interactions between these two modifiable factors, offering novel insights into their combined impact on cognitive health. Third, we employed advanced statistical approaches including non-linear relationships detection and robust adjustment for confounders, addressing methodological limitations of prior studies. Our findings could inform the development of integrated preventive strategies that simultaneously target nutritional and sleep-related risk factors for cognitive decline in aging populations, particularly in rapidly developing societies experiencing substantial lifestyle and dietary transitions.

## 2 Methods

### 2.1 Study design and participants

The SHARE is an ongoing prospective cohort study established by the Public Health Research Center of Shanghai Tongren Hospital. Launched in January 2024 with support from the Health Commission of Changning District, the study draws participants from 10 community health service centers (CHSCs) and 8 clinical departments across three districts in Shanghai (Changning, Chongming, and Jinshan). Using standardized protocols, comprehensive baseline assessments collect multidimensional health information including demographics, lifestyle factors, cognitive function, and anthropometric and biochemical measurements through a secure real-time data collection platform. For the present analysis, we included baseline data collected between January and May 2024. Eligible participants were adults aged 65 years or older who: (1) had resided in the target districts for more than 1 year; (2) were free of severe physical or cognitive impairments that would preclude study participation; and (3) were capable of providing informed consent and willing to complete all study procedures. The final analytical sample comprised 8,806 participants (participant selection detailed in Supplementary Figure S1). The study protocol was approved by the ethics committee of Shanghai Tongren Hospital (K2024-019-02), and all participants provided written informed consent.

### 2.2 Sample size calculation

Sample size was calculated based on the primary outcome of cognitive impairment (MMSE score ≤ 24). According to previous studies in urban Chinese elderly populations, the prevalence of cognitive impairment ranges from 15.4% to 27.5% (23–25). Assuming a conservative prevalence estimate of 15%, an odds ratio of 1.2 for the association between exposure (B-complex vitamins intake or sleep quality) and cognitive impairment, a two-sided alpha of 0.05, and 90% power, we estimated a minimum required sample size of 4,276 participants. To account for potential missing data (estimated at 20%) and enable robust interaction analyses requiring larger sample sizes, we aimed to recruit approximately 5,345 participants. The final enrolled sample of 8,806 participants exceeded this target, providing adequate power for both main effects and interaction analyses.

### 2.3 Data collection and quality control

Data collection was conducted through a secure, centralized digital platform developed by the Public Health Research Center of Shanghai Tongren Hospital. This platform implemented a three-tier access control system: full administrative privileges for the core research team, restricted operational permissions for participating centers, and participant-specific access through QR code-based authentication. Participants initiated data entry by scanning a unified QR code, selecting their affiliated health center, and completing identity verification using their national identification number. The platform incorporated multiple quality control features: (1) real-time data validation with predefined acceptable ranges for all variables; (2) standardized photographic aids for accurate dietary portion size estimation; and (3) automated logic checks for data consistency. Quality assurance was further enhanced through weekly systematic audits by the research team, who reviewed data completeness, accuracy, and consistency across all 18 participating centers (10 CHSCs and 8 clinical departments). Centers received detailed feedback reports and, when necessary, additional training to maintain data quality standards.

### 2.4 Measurements

#### 2.4.1 B-complex vitamins intake assessment

Dietary intake was evaluated using a validated Food Frequency Questionnaire (FFQ) specifically adapted for the Chinese population. The FFQ captured consumption patterns of 18 major food groups that characterize traditional and modern Chinese dietary habits. For each food group, participants reported both frequency (daily, weekly, or monthly) and quantity (in standardized portions and grams) of consumption over the preceding 12 months. To enhance the accuracy of portion size estimation, trained staff used a standardized visual aid handbook containing life-size photographs of common utensils and reference objects to help participants visually estimate and report their typical portion sizes for each food group. Daily intake of B-complex vitamins (B6, B12, and folate) was calculated using the China Food Composition Database, Second Edition (2004) (26) and the NutriData Platform (Chinese Nutrition Society) (27). The FFQ's validity and reliability have been previously established in multiple Chinese population-based studies, showing good correlation with 24-h dietary recalls (correlation coefficients: 0.17–0.59) and acceptable reproducibility (intraclass correlation coefficients: 0.19–0.53) (28–30). To integrate the three vitamins into a composite score, the vitamin B index was calculated as the arithmetic mean of the standardized values (z-scores) of daily intakes for vitamin B6, vitamin B12, and folate, providing a composite measure of overall B-complex vitamins status. For interaction analyses, we categorized vitamin B index using population-specific quartiles, with “low intake” defined as ≤ 50th percentile and “high intake” as >50th percentile of the distribution.

#### 2.4.2 Sleep quality assessment

Sleep quality was evaluated using the Pittsburgh Sleep Quality Index (PSQI) (31), a comprehensive sleep assessment tool comprising 19 self-rated items across seven domains: (1) subjective sleep quality, (2) sleep latency, (3) sleep duration, (4) habitual sleep efficiency, (5) sleep disturbances, (6) use of sleep medications, and (7) daytime dysfunction. Each domain is scored from 0 to 3, yielding a global PSQI score ranging from 0 to 21, with higher scores indicating poorer sleep quality. The Chinese PSQI has demonstrated robust psychometric properties in various populations, including satisfactory internal consistency (Cronbach's α = 0.68–0.78) and test-retest reliability (*r* = 0.73–0.81) among patients with type 2 diabetes (32), community-dwelling centenarians (33), and frontline COVID-19 health care workers (34). For interaction analyses, we classified sleep quality using the 75th percentile of PSQI scores as the threshold, defining “poor sleep quality” as PSQI >50th percentile and “good sleep quality” as PSQI ≤ 50th percentile.

#### 2.4.3 Cognitive function assessment

Cognitive function was evaluated using the Chinese version of the Mini-Mental State Examination (MMSE) (35), a widely-used screening tool that assesses six cognitive domains: (1) temporal orientation, (2) spatial orientation, (3) registration and recall, (4) attention and calculation, (5) language ability, and (6) visuospatial construction. The MMSE yields a total score ranging from 0 to 30, with higher scores indicating better cognitive performance. We defined probable cognitive impairment using a cutoff score of ≤ 24, which has been validated against neuroimaging markers in aging populations. This threshold demonstrates strong correlation with hippocampal atrophy, a recognized biomarker of pathological aging and neurodegeneration (36). The Chinese version of MMSE has shown robust measurement properties in adults aged ≥65 years, with a validated seven-factor structure showing strict factorial invariance across age groups and high reliability (Zumbo's α > 0.89 for all domains) (37).

#### 2.4.4 Covariates

Analyses adjusted for comprehensive sets of potential confounding variables including sociodemographic characteristics, lifestyle factors, and health status indicators. Sociodemographic variables comprised age (continuous), gender (male/female), education level (primary school/middle or high school/bachelor's degree/master's degree), marital status (never married/married/divorced or widowed), living arrangement (alone/not alone), retirement status (yes/no), occupation history (professionals or government staff/others), and monthly household income (≤ 5,000/5,001–9,999/10,000–19,999/≥20,000 RMB). Lifestyle factors included smoking status (yes or no), alcohol consumption (yes or no), tea or coffee consumption (yes/no), dietary preferences (light taste/other), cooking oil type (animal/herbal vegetable/woody vegetable/other), dietary supplement use (vitamins/calcium/none), physical activity (weekly metabolic equivalent task-hours, continuous), entertainment and social engagement frequency (never/monthly/weekly/daily). Health status was assessed through hypertension status (yes or no), diabetes status (yes or no), dyslipidemia status (yes or no), heart disease status (yes or no), stroke status (yes or no), total chronic disease burden (number of physician-diagnosed conditions) and Body Mass Index (BMI, kg/m^2^, calculated from measured height and weight).

### 2.5 Statistical analysis

All analyses were performed using R version 4.5.1 (R Foundation for Statistical Computing, Vienna, Austria). Continuous variables were summarized as means ± standard deviations and categorical variables as frequencies with percentages. Between-group comparisons by cognitive status used Kruskal-Wallis tests for continuous variables and Chi-squared tests for categorical variables.

We employed a data-driven approach for covariate selection using the Least Absolute Shrinkage and Selection Operator (LASSO) regression with 10-fold cross-validation. This yielded two sets of covariates based on the one standard error (SE) and minimum lambda criteria. Three sequential models were constructed: an unadjusted model (model 1), a parsimonious model adjusted for covariates selected by the one SE criterion (model 2), and a fully adjusted model including all covariates identified by the minimum criterion (model 3). Restricted cubic splines (RCS) with 4 knots were applied to examine potential non-linear relationships between B-complex vitamins intake and sleep quality with cognitive function. Independent associations of B-complex vitamins intake and sleep quality with cognitive function were assessed using multivariate logistic regression analysis.

We assessed potential interactions between B-complex vitamins intake and sleep quality using both multiplicative and additive scales. Multiplicative interaction was evaluated by including cross-product terms in the logistic regression models. For additive interaction, we calculated three measures with 95% confidence intervals: the relative excess risk due to interaction (RERI), the attributable proportion due to interaction (AP), and the synergy index (SI). To further characterize joint effects, participants were stratified into four groups based on B-complex vitamins intake (high: >50th percentile; low: ≤ 50th percentile) and sleep quality (good: PSQI ≤ 50th percentile; poor: PSQI >50th percentile). Statistical significance was set at a two-tailed *P-value* < 0.05. To ensure the robustness of the exposure classifications, we conducted sensitivity analyses using alternative percentile thresholds (25th/75th, 33.3th/66.7th, and 75th/25th percentiles) for defining low or high B-complex vitamins intake and poor or good sleep quality.

## 3 Results

### 3.1 Baseline characteristics

Among the 8,806 participants, 677 (7.69%) met the criteria for cognitive impairment. The mean daily intake of B-complex vitamins was 0.80 mg for vitamin B6, 5.06 μg for vitamin B12, and 263.38 μg for folate. The mean PSQI score was 5.87, indicating generally moderate sleep quality in this population. Participants with cognitive impairment differed significantly from those without impairment across multiple domains. They were more likely to be older, female, have lower educational attainment, report lower household income, be divorced or widowed, and live alone. Their lifestyle patterns showed lower rates of alcohol, tea, and coffee consumption, higher use of animal-based cooking oils, and reduced engagement in physical activity, entertainment, and social activity. Additionally, they exhibited a greater burden of chronic diseases, lower intake of B-complex vitamins, and poorer sleep quality as indicated by higher PSQI scores (all *P* < 0.05; detailed characteristics presented in Table 1).

**Table 1:** Baseline characteristics of participants overall and comparison by cognitive impairment status.

**Variables^a^**	**Total**	**Cognitive impairment status**		***P*-value^b^**
	***N*** = **8,806**	**No (*****N*** = **8,129)**	**Yes (** ***N*** = **677)**	
Age	73.16 (6.06)	72.90 (5.89)	76.39 (7.12)	<0.001
**Gender**				0.001
Male	3,733 (42.39%)	3,489 (42.92%)	244 (36.04%)	
Female	5,073 (57.61%)	4,640 (57.08%)	433 (63.96%)	
**Education level**				<0.001
Primary school	793 (9.01%)	633 (7.79%)	160 (23.63%)	
Middle/High school	6,914 (78.51%)	6,474 (79.64%)	440 (64.99%)	
Bachelor's degree	1,091 (12.39%)	1,015 (12.49%)	76 (11.23%)	
Master's degree	8 (0.09%)	7 (0.09%)	1 (0.15%)	
**Marital status**				<0.001
Never married	60 (0.68%)	53 (0.65%)	7 (1.03%)	
Married	7,745 (87.95%)	7,228 (88.92%)	517 (76.37%)	
Divorced or widowed	1,001 (11.37%)	848 (10.43%)	153 (22.60%)	
**Living status**				<0.001
Alone	838 (9.52%)	737 (9.07%)	101 (14.92%)	
Not alone	7,968 (90.48%)	7,392 (90.93%)	576 (85.08%)	
**Whether retire**				0.114
Yes	8,622 (97.91%)	7,953 (97.83%)	669 (98.82%)	
No	184 (2.09%)	176 (2.17%)	8 (1.18%)	
**Career**				0.648
Professionals/Government staff	1,537 (17.45%)	1,414 (17.39%)	123 (18.17%)	
Other	7,269 (82.55%)	6,715 (82.61%)	554 (81.83%)	
**Family income**				<0.001
≤ 5,000	864 (9.81%)	760 (9.35%)	104 (15.36%)	
5,001–9,999	4,997 (56.75%)	4,669 (57.44%)	328 (48.45%)	
10,000–19,999	2,606 (29.59%)	2,376 (29.23%)	230 (33.97%)	
≥ 20,000	339 (3.85%)	324 (3.99%)	15 (2.22%)	
**Smoking status** ^c^				0.406
Yes	745 (8.46%)	694 (8.54%)	51 (7.53%)	
No	8,061 (91.54%)	7,435 (91.46%)	626 (92.47%)	
**Drinking status** ^c^				<0.001
Yes	825 (9.37%)	788 (9.69%)	37 (5.47%)	
No	7,981 (90.63%)	7,341 (90.31%)	640 (94.53%)	
**Drink tea**				0.002
Yes	2,342 (26.60%)	2,196 (27.01%)	146 (21.57%)	
No	6,464 (73.40%)	5,933 (72.99%)	531 (78.43%)	
**Drink coffee**				0.001
Yes	845 (9.60%)	805 (9.90%)	40 (5.91%)	
No	7,961 (90.40%)	7,324 (90.10%)	637 (94.09%)	
**Taste** ^c^				0.461
Light	6,062 (68.84%)	5,605 (68.95%)	457 (67.50%)	
Other	2,744 (31.16%)	2,524 (31.05%)	220 (32.50%)	
**Oil type** ^c^				<0.001
Animal oil	90 (1.02%)	69 (0.85%)	21 (3.10%)	
Herbal vegetable oil	8,020 (91.07%)	7,422 (91.30%)	598 (88.33%)	
Woody vegetable oil	635 (7.21%)	586 (7.21%)	49 (7.24%)	
Other	61 (0.69%)	52 (0.64%)	9 (1.33%)	
**Vitamin supplement**				0.453
Yes	1,169 (13.28%)	1,086 (13.36%)	83 (12.26%)	
No	7,637 (86.72%)	7,043 (86.64%)	594 (87.74%)	
**Calcium supplement**				0.168
Yes	1,391 (15.80%)	1,271 (15.64%)	120 (17.73%)	
No	7,415 (84.20%)	6,858 (84.36%)	557 (82.27%)	
Weekly MET-hours	469.18 (653.59)	479.00 (644.77)	351.17 (741.91)	<0.001
**Entertainment**				<0.001
Never	1,179 (13.39%)	976 (12.01%)	203 (29.99%)	
At least once a month	540 (6.13%)	467 (5.74%)	73 (10.78%)	
At least once a week	705 (8.01%)	640 (7.87%)	65 (9.60%)	
Almost everyday	6,382 (72.47%)	6,046 (74.38%)	336 (49.63%)	
**Social activity**				<0.001
Never	5,925 (67.28%)	5,404 (66.48%)	521 (76.96%)	
At least once a month	1,945 (22.09%)	1,821 (22.40%)	124 (18.32%)	
At least once a week	713 (8.10%)	682 (8.39%)	31 (4.58%)	
Almost everyday	223 (2.53%)	222 (2.73%)	1 (0.15%)	
BMI, kg/m^2^	23.95 (3.27)	23.93 (3.27)	24.19 (3.27)	0.052
**Hypertension**				<0.001
Yes	5,429 (61.65%)	4,946 (60.84%)	483 (71.34%)	
No	3,377 (38.35%)	3,183 (39.16%)	194 (28.66%)	
**Diabetes**				<0.001
Yes	2,352 (26.71%)	2,127 (26.17%)	225 (33.23%)	
No	6,454 (73.29%)	6,002 (73.83%)	452 (66.77%)	
**Dyslipidemia**				<0.001
Yes	1,266 (14.38%)	1,125 (13.84%)	141 (20.83%)	
No	7,540 (85.62%)	7,004 (86.16%)	536 (79.17%)	
**Heart disease**				0.045
Yes	1,056 (11.99%)	958 (11.78%)	98 (14.48%)	
No	7750 (88.01%)	7171 (88.22%)	579 (85.52%)	
**Stroke**				<0.001
Yes	345 (3.92%)	295 (3.63%)	50 (7.39%)	
No	8,461 (96.08%)	7,834 (96.37%)	627 (92.61%)	
**Number of diseases**				<0.001
0	1,774 (20.15%)	1,693 (20.83%)	81 (11.96%)	
1–2	5,742 (65.21%)	5,310 (65.32%)	432 (63.81%)	
≥3	1,290 (14.65%)	1,126 (13.85%)	164 (24.22%)	
Daily vitamin B6 intake, mg	0.80 (0.43)	0.82 (0.43)	0.62 (0.35)	<0.001
Daily vitamin B12 intake, μg	5.06 (3.02)	5.14 (3.04)	4.11 (2.50)	<0.001
Daily folate intake, μg	263.38 (142.88)	268.27 (143.66)	204.58 (118.21)	<0.001
PSQI score	5.87 (3.11)	5.76 (3.04)	7.27 (3.56)	<0.001

MET, metabolic equivalent of task; BMI, body mass index; PSQI, Pittsburgh sleep quality index.

^a^Continuous variables were presented as the mean ± standard deviation (SD); Categorical variables were presented as frequency and percentage.

^b^For continuous variables, P-values for difference by cognitive impairment status were calculated by the Kruskal-Wallis test; for categorical variables, P-values were calculated by the chi-square test.

^c^The variables “Smoking status” and “Drinking status” were defined as the response to the question “Do you currently smoke/drink alcohol?”; The variable “Taste” was defined as the response to the question “What is your primary taste preference?”; The variable “Oil type” was defined as the response to the question “What is the most commonly used cooking oil in your household?.”

### 3.2 Covariate selection using LASSO regression

We employed LASSO regression with 10-fold cross-validation to systematically identify key predictors of cognitive impairment from 26 baseline covariates. The coefficient trajectories demonstrated variable shrinkage patterns across the spectrum of lambda values (Supplementary Figure S2A). Two optimal regularization parameters were determined: the one-standard-error (1-SE) criterion (λ = 0.027, log λ = −3.60) prioritized model parsimony while maintaining predictive accuracy, retaining six core variables (age, education level, marital status, entertainment frequency, social activity level, and number of chronic diseases); the minimum lambda criterion (λ = 0.005, log λ = −5.37) maximized prediction performance, selecting an extended set of 19 variables that additionally included gender, retirement status, career type, income level, smoking status, alcohol consumption, coffee consumption, taste preference, calcium supplementation, hypertension, dyslipidemia, heart disease, and stroke (Supplementary Figure S2B, Supplementary Tables S1, S2). Both variable sets were subsequently incorporated into separate multivariate logistic regression models to evaluate the robustness of associations under different levels of covariate adjustment. The results of multicollinearity tests indicated no significant correlation among two exposure factors and covariates included in multivariate logistic regression models (the VIF for all the factors was <5) (Supplementary Table S3).

### 3.3 Independent associations of B-complex vitamins intake and sleep quality with cognitive function

**Figure 1:**
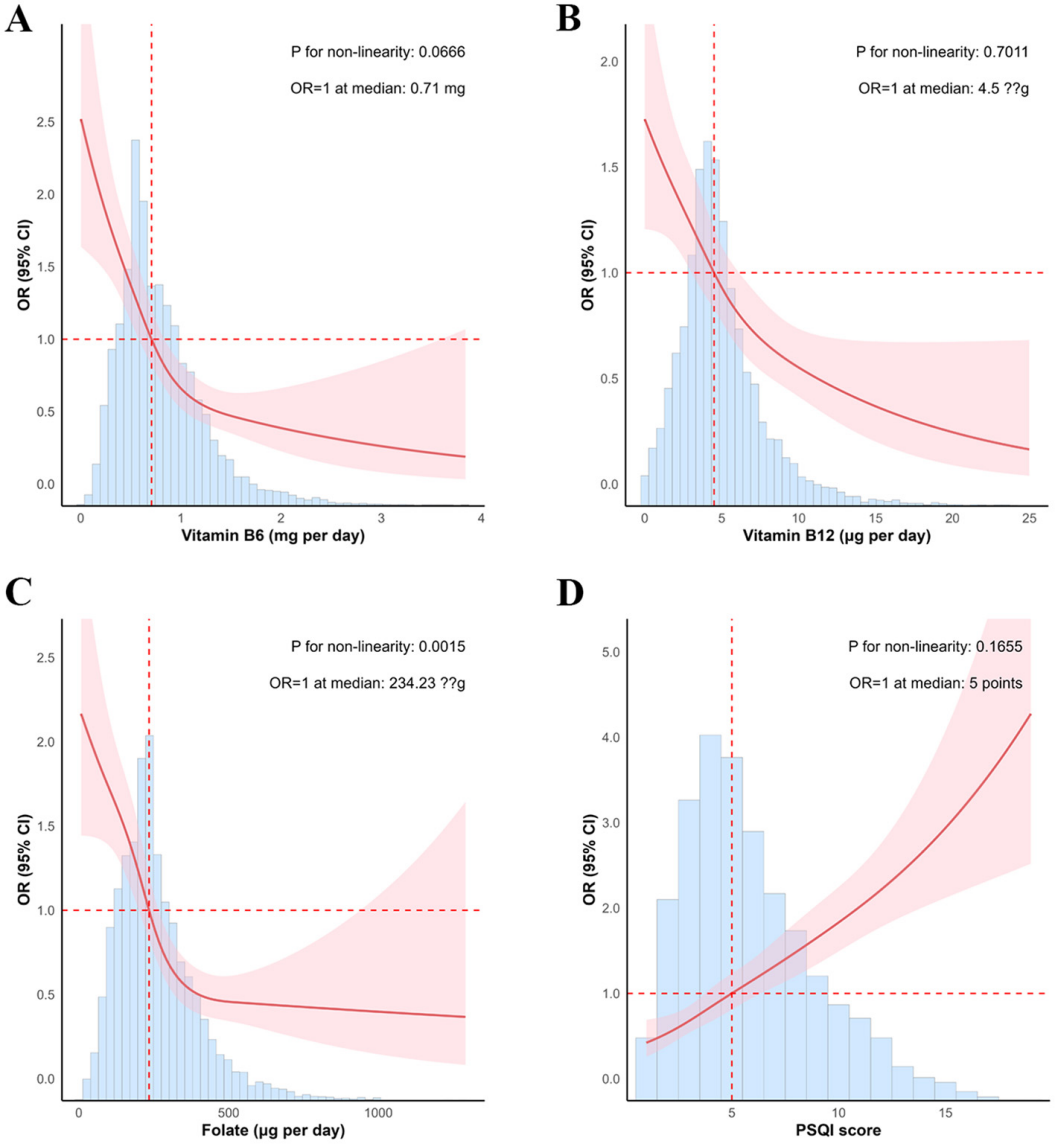
The restricted cubic splines for the associations of B-complex vitamins intake and sleep quality with cognitive function. Adjusted for age, education, marriage, entertainment, social, number of diseases, hypertension, dyslipidemia, heart disease, stroke, gender, retire status, career, income, smoke, drink, coffee, taste, and calcium supplement status. B-complex vitamins in **(A–C)**, sleep quality in **(D)**.

RCS analyses in the fully adjusted model (Model 3) revealed distinct dose-response patterns between cognitive impairment risk and both B-complex vitamins intake and sleep quality. B-vitamin associations demonstrated protective L-shaped relationships, with differential patterns across vitamins. Vitamin B6 and B12 intake showed predominantly linear inverse associations with cognitive impairment risk (P for non-linearity = 0.058 and 0.681, respectively), while folate intake exhibited a significant non-linear relationship (P for non-linearity = 0.002). Sleep quality, assessed by PSQI scores, demonstrated a J-shaped relationship with cognitive impairment risk (P for non-linearity = 0.191), indicating progressively increased risk with poorer sleep quality. These dose-response patterns remained robust across sensitivity analyses using different covariate adjustment strategies (Figure 1, Supplementary Figures S3, S4).

Multivariate logistic regression analyses corroborated the RCS findings, demonstrating significant associations between cognitive impairment risk and both B-complex vitamins intake and sleep quality across all adjustment models. Quartile categorization of exposures were used to examine threshold effects. In the fully adjusted model (Model 3), using the first quartile (Q1) as reference, higher B-vitamin intake demonstrated progressively stronger protective effects. For vitamin B6, the odds ratios (ORs) [95% confidence intervals (CIs)] were 0.86 [0.69, 1.06] (*P* = 0.160), 0.53 [0.42, 0.67] (*P* < 0.001), and 0.33 [0.25, 0.43] (*P* < 0.001) for Q2–Q4, respectively. Similarly, vitamin B12 and folate exhibited comparable protective dose-response patterns, with the highest quartile (Q4) showing the most substantial risk reduction. Conversely, poorer sleep quality showed dose-dependent adverse effects, with ORs of 1.38 [1.04, 1.82] (*P* = 0.023), 1.92 [1.54, 2.40] (*P* < 0.001), and 2.94 [2.34, 3.69] (*P* < 0.001) for Q2–Q4, respectively (Table 2).

**Table 2:** Independent associations of B-complex vitamins intake and sleep quality with cognitive function by logistic regression analysis.

**Variables**		**Model 1** ^**a**^		**Model 2** ^**b**^		**Model 3** ^**c**^	
		**OR [95% CI]**	***P*** **-value**	**OR [95% CI]**	***P*** **-value**	**OR [95% CI]**	***P*** **-value**
VB6	Q1	Reference		Reference		Reference	
	Q2	0.61 [0.50, 0.74]	<0.001	0.89 [0.72, 1.10]	0.285	0.86 [0.69, 1.06]	0.160
	Q3	0.42 [0.34, 0.52]	<0.001	0.55 [0.44, 0.69]	<0.001	0.53 [0.42, 0.67]	<0.001
	Q4	0.25 [0.20, 0.33]	<0.001	0.34 [0.26, 0.45]	<0.001	0.33 [0.25, 0.43]	<0.001
VB12	Q1	Reference		Reference		Reference	
	Q2	0.61 [0.49, 0.74]	<0.001	0.75 [0.60, 0.93]	0.008	0.72 [0.58, 0.90]	0.003
	Q3	0.52 [0.42, 0.64]	<0.001	0.67 [0.53, 0.83]	<0.001	0.64 [0.51, 0.81]	<0.001
	Q4	0.36 [0.28, 0.45]	<0.001	0.45 [0.35, 0.58]	<0.001	0.43 [0.33, 0.55]	<0.001
Folate	Q1	Reference		Reference		Reference	
	Q2	0.60 [0.49, 0.73]	<0.001	0.81 [0.66, 1.00]	0.051	0.78 [0.64, 0.97]	0.024
	Q3	0.31 [0.24, 0.39]	<0.001	0.42 [0.32, 0.53]	<0.001	0.40 [0.31, 0.51]	<0.001
	Q4	0.27 [0.21, 0.34]	<0.001	0.35 [0.27, 0.45]	<0.001	0.33 [0.25, 0.43]	<0.001
PSQI score	Q1	Reference		Reference		Reference	
	Q2	1.35 [1.03, 1.77]	0.028	1.41 [1.06, 1.85]	0.016	1.38 [1.04, 1.82]	0.023
	Q3	1.77 [1.44, 2.19]	<0.001	1.95 [1.57, 2.43]	<0.001	1.92 [1.54, 2.40]	<0.001
	Q4	2.89 [2.34, 3.56]	<0.001	2.94 [2.36, 3.69]	<0.001	2.94 [2.34, 3.69]	<0.001

VB6, vitamin B6; VB12, vitamin B12; PSQI, Pittsburgh sleep quality index; OR, odds ratios; CI, confidence interval; Q1–Q4, 1st quartile-4th quartile.

^a^Model 1: crude model.

^b^Model 2: adjusted for age, education, marriage, entertainment, social, and number of diseases.

^c^Model 3: additionally adjusted for hypertension, dyslipidemia, heart disease, stroke, gender, retire status, career, income, smoke, drink, coffee, taste, and calcium supplement status.

### 3.4 Multiplicative and additive interactions between B-complex vitamins intake and sleep quality on cognitive function

The multiplicative interaction analysis which incorporated cross-product terms in logistic regression models did not demonstrate statistically significant interactions in any of the three models examined (model 1: *P* = 0.919; model 2: *P* = 0.689; model 3: *P* = 0.710) (Table 3).

**Table 3:** Multiplicative interactions between B-complex vitamins intake and sleep quality on cognitive function.

**Variables**	**Model 1** ^**a**^		**Model 2** ^**b**^		**Model 3** ^**c**^	
	**OR [95% CI]**	***P*** **-value**	**OR [95% CI]**	***P*** **-value**	**OR [95% CI]**	***P*** **-value**
VB index	0.52 [0.40, 0.66]	<0.001	0.59 [0.47, 0.75]	<0.001	0.57 [0.45, 0.73]	<0.001
PSQI score	1.14 [1.11, 1.17]	<0.001	1.14 [1.11, 1.17]	<0.001	1.14 [1.11, 1.17]	<0.001
VB index: PSQI score	1.00 [0.97, 1.03]	0.919	1.01 [0.98, 1.04]	0.689	1.01 [0.97, 1.04]	0.710

VB6, vitamin B6; VB12, vitamin B12; PSQI, Pittsburgh sleep quality index; OR, odds ratios; CI, confidence interval; Q1-Q4, 1st quartile-4th quartile.

^a^Model 1: crude model.

^b^Model 2: adjusted for age, education, marriage, entertainment, social, and number of diseases.

^c^Model 3: additionally adjusted for hypertension, dyslipidemia, heart disease, stroke, gender, retire status, career, income, smoke, drink, coffee, taste, and calcium supplement status.

To further investigate domain-specific interactions, stratified analyses across the seven PSQI components were conducted. Significant multiplicative interactions were observed for three specific domains: subjective sleep quality (OR=0.67, 95% CI: 0.51–0.86; *P* = 0.002), sleep disturbances (OR = 1.45, 95% CI: 1.15–1.81; *P* = 0.002), and daytime dysfunction (OR = 1.22, 95% CI: 1.06–1.40; *P* = 0.004) in model 3. However, no significant interactions were detected for sleep latency, sleep duration, sleep efficiency, or use of sleep medications (Supplementary Table S4).

Additive interaction analyses in the fully adjusted model (Model 3) revealed significant synergistic effects between low B-complex vitamins intake and poor sleep quality on cognitive impairment risk. The Relative Excess Risk due to Interaction (RERI) was 0.97 [95% CI: 0.48–1.46, *P* < 0.001], indicating that the combined exposure increased cognitive impairment risk by 0.97 units beyond additive effects, representing a 28.7% higher risk than expected (observed OR: 4.35 vs. expected OR: 3.38). The Attributable Proportion due to interaction (AP) was 0.22 [95% CI: 0.10–0.35, *P* < 0.001], suggesting that 22% of cognitive impairment risk among individuals with both exposures was specifically attributable to their interaction. The Synergy Index (SI) of 1.41 [95% CI: 1.14–1.67, *P* < 0.001] further confirmed super-additive effects. These interaction measures remained consistent across all adjustment models, providing robust evidence for biological interaction despite the absence of multiplicative effects (Table 4).

**Table 4:** Additive interactions between B-complex vitamins intake and sleep quality on cognitive function.

**Additive interaction measures**	**Model 1** ^**a**^		**Model 2** ^**b**^		**Model 3** ^**c**^	
	**Value [95% CI]**	***P*** **-value**	**Value [95% CI]**	***P*** **-value**	**Value [95% CI]**	***P*** **-value**
RERI	1.03 [0.56, 1.50]	<0.001	0.94 [0.46, 1.42]	<0.001	0.97 [0.48, 1.46]	<0.001
AP	0.22 [0.11, 0.34]	<0.001	0.22 [0.09, 0.35]	<0.001	0.22 [0.10, 0.35]	<0.001
SI	1.40 [1.17, 1.64]	<0.001	1.41 [1.14, 1.69]	<0.001	1.41 [1.14, 1.67]	<0.001

RERI, relative excess risk due to interaction; AP, attributable proportion; SI, synergy index; CI, confidence, interval.

^a^Model 1: crude model.

^b^Model 2: adjusted for age, education, marriage, entertainment, social, and number of diseases.

^c^Model 3: additionally adjusted for hypertension, dyslipidemia, heart disease, stroke, gender, retire status, career, income, smoke, drink, coffee, taste, and calcium supplement status.

**Figure 2:**
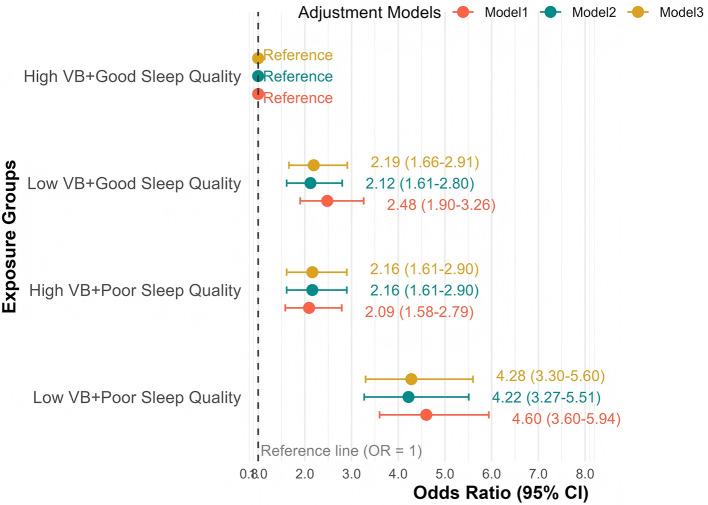
Cognitive impairment risk by B-complex vitamins intake and sleep quality status.

Stratified analyses in the fully adjusted model (Model 3) demonstrated distinct risk patterns across exposure combinations. Taking participants with high B-complex vitamins intake and good sleep quality as the reference (group 1), participants with concurrent low B-complex vitamins intake and poor sleep quality (group 2) exhibited the highest cognitive impairment risk [OR: 4.35 (95% CI: 3.35–5.70), *P* < 0.001], substantially exceeding the risks associated with isolated exposures to either low vitamins intake (group 3) [OR: 2.21 (95% CI: 1.67–2.93), *P* < 0.001] or poor sleep quality (group 4) [OR: 2.17 (95% CI: 1.62–2.92), *P* < 0.001]. This risk pattern remained consistent across all adjustment models, reinforcing the presence of additive effects despite the absence of multiplicative interaction (Figure 2, Supplementary Table S5).

Sensitivity analyses using alternative percentile thresholds demonstrated consistent findings, with all threshold combinations showing significant additive interactions (Supplementary Table S6). To enhance the public health interpretability, we calculated the absolute risk (AR) of cognitive impairment across the exposure groups. In model 3, the AR [95% CI] was 2.59% [2.07, 3.24] in the reference group (group 1). In comparison, it increased to 5.55% [4.70, 6.53] [risk difference (RD) = 2.96%] for group 2, 5.45% [4.53, 6.54] (RD = 2.86%) for group 3, and 10.37% [9.11, 11.77] (RD = 7.78%) for group 4. The RD for the combined exposure exceeded the sum of the RDs for the individual exposures (2.96% + 2.86% = 5.82% vs. observed 7.78%), demonstrating additive interaction on the absolute risk scale (Supplementary Table S7).

## 4 Discussion

This study revealed significant independent and synergistic associations between B-complex vitamins intake and sleep quality with cognitive impairment in Chinese older adults. We identified that combined exposure to suboptimal B-complex vitamins intake and poor sleep quality was associated with a greater cognitive risk than either factor alone, with evidence of significant additive interaction effects.

Our analyses yielded three principal quantitative findings. First, vitamin B6, B12, and folate intake demonstrated L-shaped dose-response relationships with cognitive function, suggesting that cognitive benefits plateaued once nutritional requirements were met. This result closely aligned with China's Dietary Reference Intakes (DRIs). The recommended Adequate Intake (AI) of vitamin B6 and B12, and the Recommended Nutrient Intake (RNI) of folate for older adults over 50 years are 1.5 mg/day, 2.4 μg/day, and 400 μg/day (26). Our findings suggest that cognitive benefits stabilize near these reference values, supporting the scientific rationale behind current nutritional guidelines. Second, sleep quality exhibited a J-shaped association with cognitive impairment, with risk increasing progressively at higher PSQI scores. This finding demonstrated that rather than merely exceeding conventional PSQI cut-off values (typically ranging from 5 to 11) (31, 38, 39), any enhancement in sleep quality may yield additional benefits for cognitive preservation in older adults. This suggested that for optimal cognitive health, the objective should extend beyond avoiding “poor sleep” as defined by these cut-offs, to actively pursuing the highest attainable sleep quality. This finding was consistent with a study of Chinese community-dwelling older adults, which also reported a positive linear relationship between sleep quality and cognitive scores (14). In addition, a prospective study using data from UK Biobank (UKB) further supported the link between sleep and cognitive function by finding that longer weekend recovery sleep showed a linear association with reduced all-cause dementia risk in individuals with suboptimal weekday sleep (40). Third, additive interaction analyses revealed that concurrent exposure to low B-complex vitamins intake and poor sleep quality conferred a 28% higher risk than expected from their individual effects, indicating biological synergy. These findings support two key implications: adequate B-vitamins intake was associated with a threshold of cognitive benefit, while optimal sleep quality showed a continuous association with cognitive benefits in aging populations.

Previous studies had established independent associations of B-complex vitamins and sleep quality with cognitive function, which were consistent with the present results. Large-scale epidemiological studies have demonstrated that adequate intake of B-complex vitamins, particularly in genetically susceptible individuals, significantly reduced AD risk (8), and a meta-analysis study confirmed their cognitive-preserving effects in elderly populations living without dementia (9). Nevertheless, subtle differences emerged in the dose-response relationships when compared with the findings of a UKB study (8). Though we both identified an L-shaped association for folate which indicated a clear threshold effect, they reported a more linear association for vitamins B6 and B12, contrasting with our threshold-effect findings. This divergence may be attributed to methodological variations in dietary assessment and distinct population characteristics, such as baseline nutritional status and genetic background, suggesting that the optimal intake level for cognitive benefit might be context-dependent. Concurrently, sleep disturbances have been associated with accelerated cognitive decline through multiple pathways, with epidemiological evidence showing strong associations between poor sleep quality and cognitive dysfunction from China (14, 41), Japan (42), and Switzerland (43). Notably, recent evidence indicated that the detrimental impact of poor sleep quality on cognition was particularly pronounced during a sensitive age window of 50–75 years (44), highlighting mid- to late-life as a critical period for sleep-related cognitive vulnerability. However, a critical gap remains in understanding how these two modifiable factors interact to influence cognitive health. To our knowledge, no prior study has examined the potential synergistic effects between B-complex vitamins and sleep quality on cognitive function, particularly in Chinese populations. Our study was the first comprehensive investigation of both multiplicative and additive interactions between these two factors, providing novel insights into their combined influence on cognitive impairment risk. These findings highlight the potential significance of considering both nutritional and sleep-related lifestyle factors in cognitive health researches and interventions.

The observed synergistic association between B-complex vitamins and sleep quality with cognitive function is consistent with their convergence on critical neurobiological pathways. As methyl donors or essential coenzymes for OCM, vitamin B6, B12, and folate regulate Hcy metabolism and support methylation processes, which are critical for neuronal homeostasis (6, 7). Simultaneously, poor sleep quality disrupts glymphatic clearance, promoting the accumulation of neurotoxic metabolites including amyloid-βpeptides, which compromise cognitive function such as synaptic plasticity, memory consolidation, and so on (11). In addition, B-complex vitamins intake and sleep quality may potentiate each other's detrimental effects, creating a vicious cycle of neuronal dysfunction. On the one hand, chronic sleep disturbances may compromise intestinal absorption and cellular uptake of B vitamins (45), creating a nutritional deficiency state that further exacerbates neuronal vulnerability. On the other hand, elevated Hcy levels induced by B vitamins deficiencies have been shown to drive oxidative stress and vascular endothelial dysfunction (46), which may exacerbate the neurotoxic effects of poor sleep quality (47). The additive interaction suggests that the co-occurrence of these two factors creates a cumulative burden on shared neuroprotective systems, ultimately exceeding the compensatory capacity and accelerating cognitive decline. This synergistic interaction may be particularly exacerbated in aging populations due to their progressive decline in both nutrient absorption efficiency (48) and sleep quality (10).

Our study revealed a significant additive interaction between B-complex vitamins intake and sleep quality on cognitive function, while no significant multiplicative interaction was observed. This dissociation may be attributed to several reasons. First, the different exposure-response patterns without shared rate-limiting steps of these two factors prevented them biologically potentiating each other's effects in a multiplicative manner. The L-shaped dose-response relationship observed for B vitamins indicated their neuroprotective effects plateau after nutritional requirements were met, while the J-shaped association of PSQI scores demonstrated cumulative cognitive risks with worsening sleep quality. Second, measurement errors inherent in FFQ-assessed vitamins intake and self-reported sleep quality could attenuate multiplicative interaction estimates. This error accumulation followed a multiplicative pattern, creating disproportionate bias toward the null hypothesis. Third, the statistical power for interaction testing scales to the inverse power of four, thus requiring far larger sample sizes than standard association testing (49), especially given the fact that prevalence of cognitive impairment was relatively low in the present study.

However, the domain-specific analysis revealed significant multiplicative interactions for three PSQI components: subjective sleep quality, sleep disturbances, and daytime dysfunction. This pattern can be explained by examining the distinct neurobiological mechanisms underlying each dimension. The interaction for subjective sleep quality might reflect B vitamins' role in emotional regulation through neurotransmitter synthesis. Experimental study have demonstrated that vitamin B6 deficiency disrupts serotonin signaling (50), which can amplify the negative perception of poor sleep quality. The association with sleep disturbances aligned with evidence that nocturnal awakenings disrupt glymphatic clearance of neurotoxins (11), while B vitamins support neuronal repair through methylation cycles (51). For daytime dysfunction, this finding corresponded with research showing that B vitamins deficiencies impaired mitochondrial energy production in prefrontal circuits (49), exacerbating cognitive complaints during waking hours.

The observed additive interaction on both relative and absolute risk scales had important implications for the formulation of public health interventions, contrasted with multiplicative interaction which assessed effects on relative scales only and may not directly translate to health impact on population level. Our findings demonstrated that the combined exposure to low B-complex vitamins intake and poor sleep quality was associated with a 7.78% excess risk of cognitive impairment, which substantially exceeded the sum of individual risks from isolated exposures. From a resource allocation perspective, our results suggested that prioritizing older adults with both risk factors for multifaceted interventions might represent an efficient strategy for reducing cognitive impairment burden in aging populations.

This study has several strengths. First, to our knowledge, this is the first study to innovatively investigate both multiplicative and additive interactions between B-complex vitamins intake and sleep quality on cognitive function in older adults, with the significant additive interaction generating a novel hypothesis about their synergistic association and highlighting the need for future mechanistic studies to elucidate the underlying biological pathways. Second, the rigorous covariates selection via LASSO regression minimized residual confounding, with robustness confirmed across multiple adjustment models. Third, the comprehensive sensitivity analyses using alternative percentile thresholds demonstrated consistent risk patterns and additive interaction effects, affirming that our findings were not dependent on arbitrary cutoff selections but reflected robust biological relationships. Fourth, the real-time data validation system and centralized quality control procedures minimized measurement errors.

However, several limitations should be acknowledged. First, the cross-sectional design precluded causal inference, the possibility of reverse causation must be considered, whereby cognitive impairment could be associated with poorer dietary habits and disrupted sleep patterns. Future longitudinal studies are needed to establish potential causality and temporal relationships. Second, the self-reported data on diet and sleep might introduce information bias. Biomarkers such as serum B-complex vitamins, or homocysteine levels were not available in our study constrained by the large-scale, population-based design, which prioritized broad feasibility and participant burden. However, the FFQ for dietary assessment and PSQI for sleep evaluation have been extensively used and validated in Chinese populations (28–30, 32–34). In addition, our study implemented rigorous quality control measures including real-time data validation, standardized portion-size visual aids during dietary assessment and weekly checks of data quality, which helped minimize potential measurement errors. Third, we acknowledge the potential for residual confounding, such as total energy intake. The practical challenges of obtaining accurate 24-h dietary recalls from elderly participants, who demonstrated difficulty with comprehensive dietary memory, prevented precise energy intake calculation. Nevertheless, we incorporated multiple dietary factors into covariate selection process, including tea consumption, coffee consumption, taste preference, cooking oil type, and vitamin or calcium supplement use. In future studies, we plan to implement a simplified 24-h dietary recall approach to estimate total energy intake more feasibly in this population. Fourth, the absence of significant multiplicative interaction should be interpreted with caution, as it may reflect limited statistical power rather than a true biological null effect. Replication in larger cohorts is warranted to confirm this finding in future studies. Last, while the MMSE provided a practical and widely adopted screening tool for cognitive impairment, its limitations in diagnostic specificity should be considered. Future studies incorporating biomarker or imaging-based diagnoses are needed to enhance diagnostic accuracy.

## 5 Conclusion

This study demonstrated that adequate B-complex vitamins intake and good sleep quality are independently associated with a lower risk of cognitive impairment in Chinese older adults, with their concurrent deficiency exhibiting synergistic effects that amplified cognitive risk beyond the sum of their individual contributions. These findings highlight the need for integrated interventions targeting both B-vitamins nutritional adequacy and sleep quality optimization in aging populations, particularly relevant in China given its rapidly expanding elderly population and growing dementia burden. Future research should examine whether targeted interventions addressing both factors could yield enhanced cognitive benefits compared to single-factor approaches.

## Data Availability

The raw data supporting the conclusions of this article will be made available by the authors, without undue reservation.
