# Supplementary material for: Dietary B-complex vitamins and sleep quality in relation to cognitive impairment among older adults: non-linear associations and evidence of additive interaction
Source: Front Nutr. 2025 Dec 19;12:1694999. doi: 10.3389/fnut.2025.1694999 (PMC12757219; doi:10.3389/fnut.2025.1694999)
Supplement: Supplementary file 1 [file Table_1.docx]

Supplementary Material

# Supplementary Figures


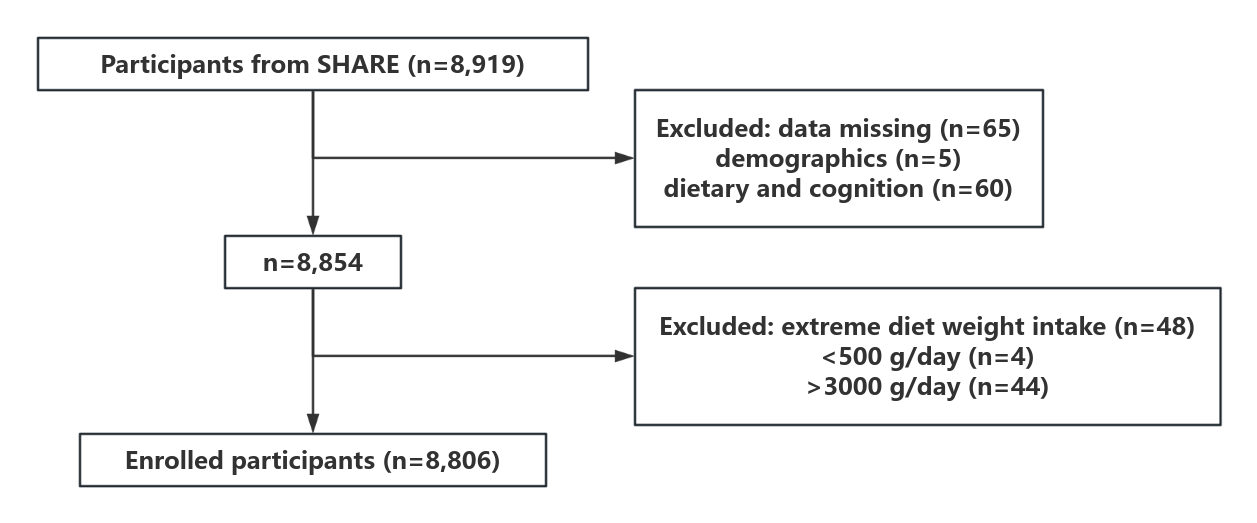


**Supplementary Figure 1.** Flowchart of participant selection process.


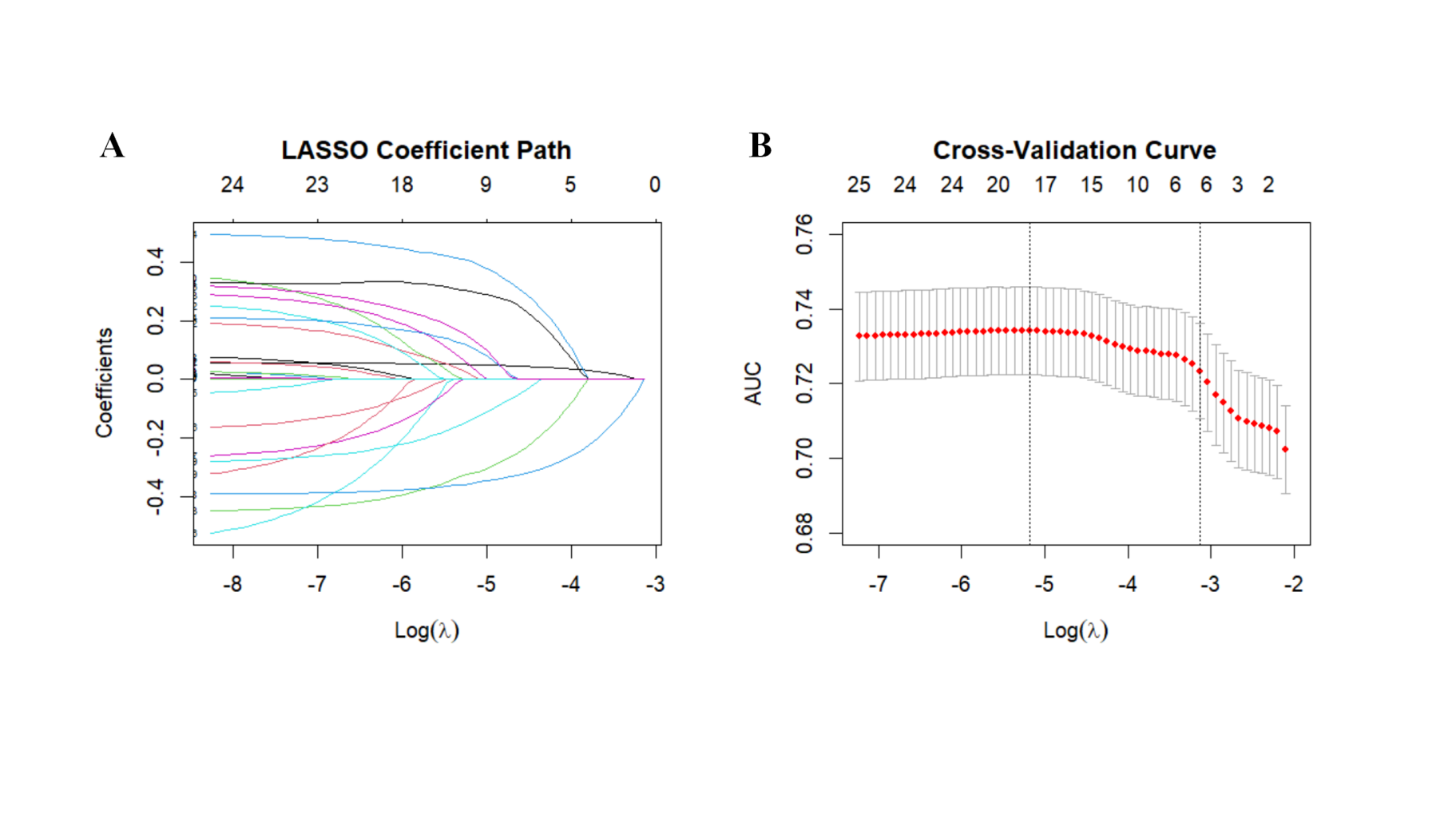


**Supplementary Figure 2.** Covariates selection based on LASSO regression. (A) Plot of LASSO coefficient profiles of the 21 candidate predictors for cognitive impairment; (B) Selection process of the optimal value of LASSO penalty parameter lambda in the Lasso regression model by 10-fold cross validation method.


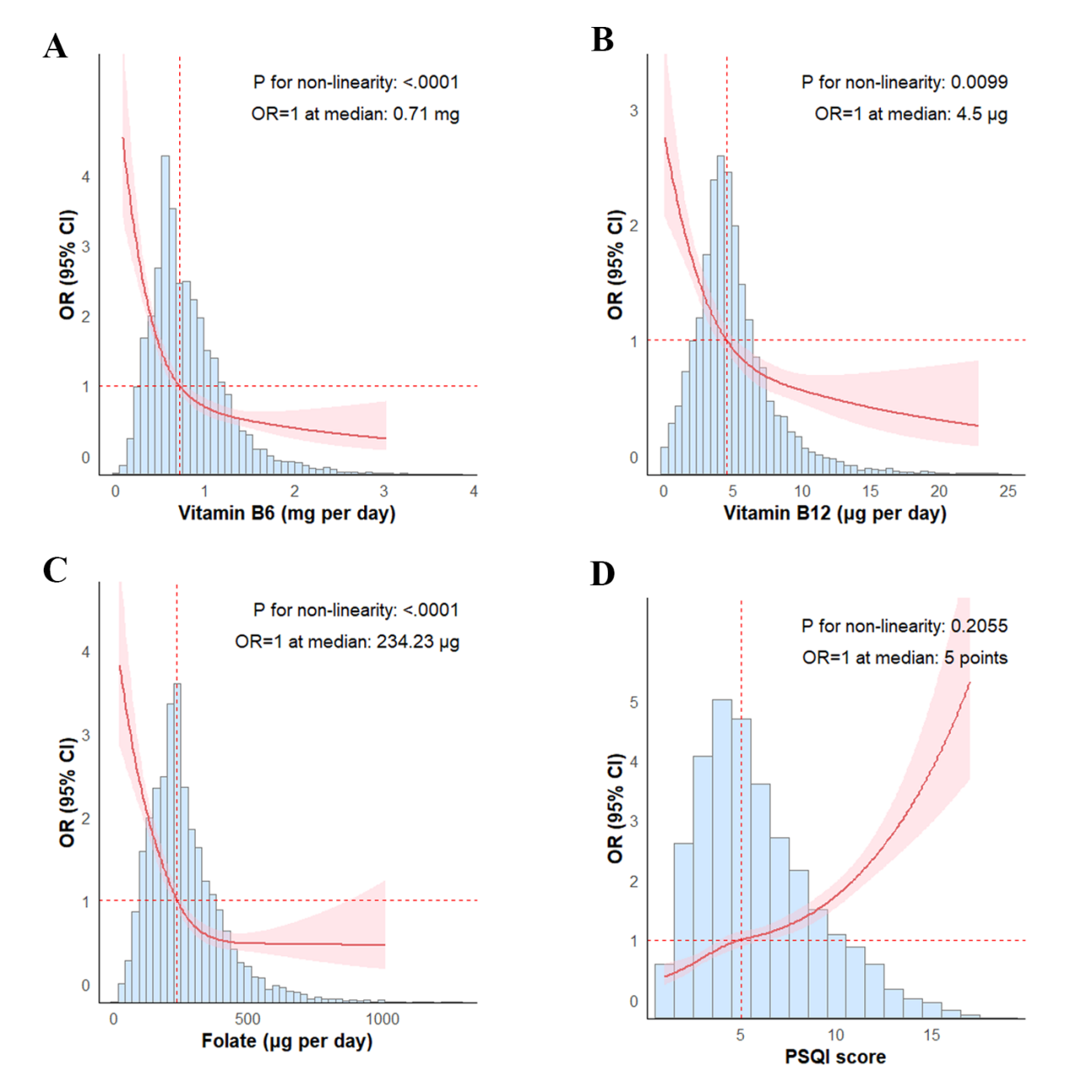


**Supplementary Figure 3.** The restricted cubic splines for the associations of B-complex vitamins intake and sleep quality with cognitive function. Crude model with no covariates adjustment. B-complex vitamins in Figure S3A-C, sleep quality in Figure S3D.


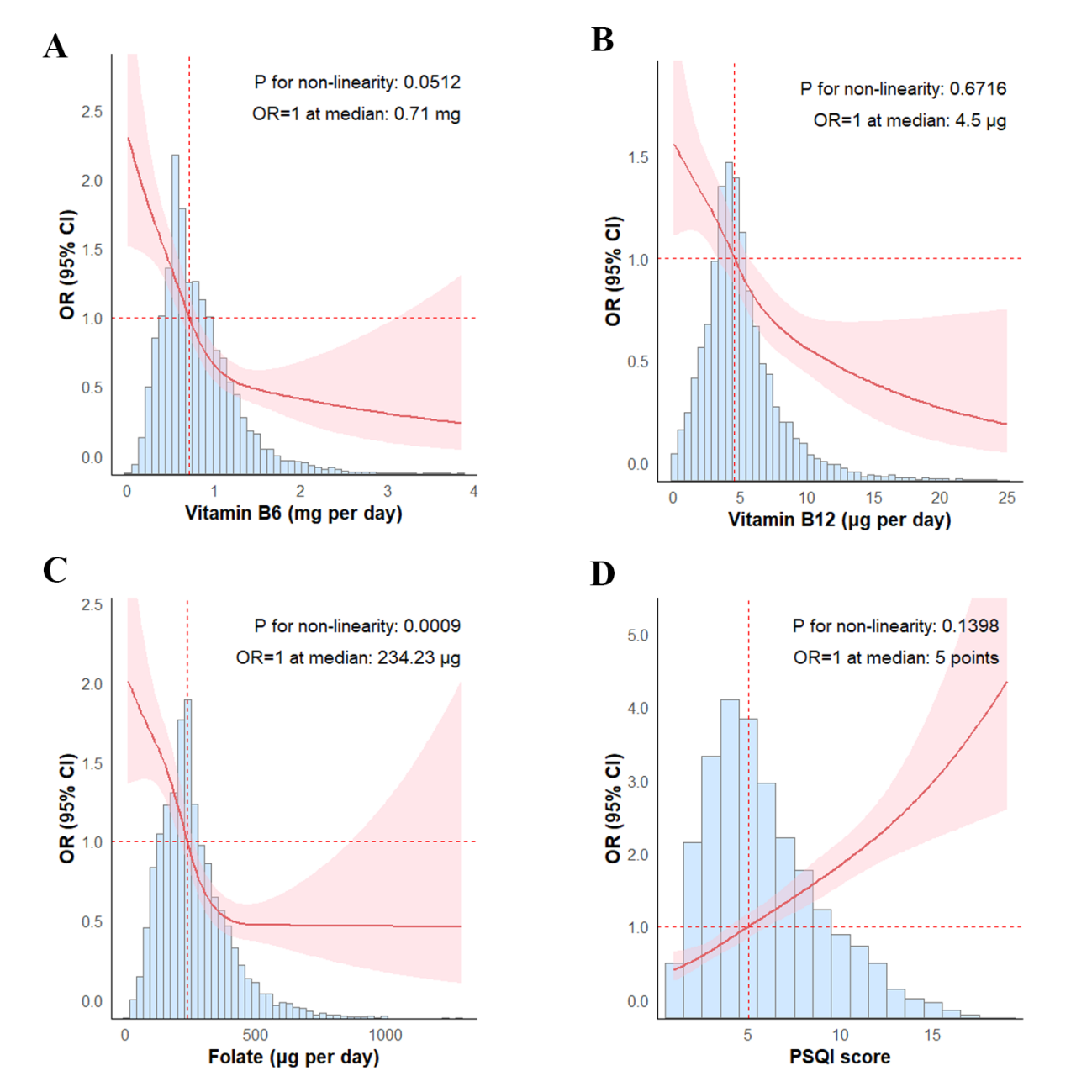


Supplementary Figure 4. The restricted cubic splines for the associations of B-complex vitamins intake and sleep quality with cognitive function. Adjusted for age, education, marriage, entertainment, social and number of diseases. B-complex vitamins in Figure S4A-C, sleep quality in Figure S4D.

# Supplementary Tables

**Supplementary Table 1.** Coefficients of the 21 candidate predictors for cognitive impairment. 6 variables with non-zero coefficients were selected using 10-fold cross validation method based on the one-standard-error (1-SE) criterion.

| **Candidate Predictors** | **Coefficients** |
| --- | --- |
| Age | 0.04740874 |
| Gender | . |
| Education level | -0.08582139 |
| Marital status | 0.08380001 |
| Living status | . |
| Whether retire | . |
| Career | . |
| Family income | . |
| Smoking status | . |
| Drinking status | . |
| Drink tea | . |
| Drink coffee | . |
| Taste | . |
| Oil type | . |
| Vitamin supplement | . |
| Calcium supplement | . |
| Weekly MET-hours | . |
| Entertainment | -0.27526867 |
| Social activity | -0.04492765 |
| BMI | . |
| Hypertension | . |
| Diabetes | . |
| Dyslipidemia | . |
| Heart disease | . |
| Stroke | . |
| Number of diseases | 0.16017900 |

**Supplementary Table 2.** Coefficients of the 26 candidate predictors for cognitive impairment. 19 variables with non-zero coefficients were selected using 10-fold cross validation method based on the minimum lambda criterion.

| **Candidate Predictors** | **Coefficients** |
| --- | --- |
| Age | 0.0689339926 |
| Gender | 0.1644543087 |
| Education level | -0.3420173363 |
| Marital status | 0.3575536596 |
| Living status | . |
| Whether retire | -0.4648585920 |
| Career | -0.2454111182 |
| Family income | 0.0006725969 |
| Smoking status | -0.1385690511 |
| Drinking status | 0.1487091393 |
| Drink tea | . |
| Drink coffee | 0.1766494054 |
| Taste | 0.2649601241 |
| Oil type | . |
| Vitamin supplement | . |
| Calcium supplement | -0.1511543389 |
| Weekly MET-hours | . |
| Entertainment | -0.4120734991 |
| Social activity | -0.2680891399 |
| BMI | . |
| Hypertension | 0.0126642151 |
| Diabetes | . |
| Dyslipidemia | 0.2091482407 |
| Heart disease | -0.0225404553 |
| Stroke | 0.3495650748 |
| Number of diseases | 0.3175054019 |

**Supplementary Table 3.** Multicollinearity test of two exposure factors and covariates included in multivariate logistic regression models using LASSO selection approach.

| **Candidate Predictors** | **GVIF** | **Df** | **Adjusted_GVIF** |
| --- | --- | --- | --- |
| VB_index | 1.092 | 1 | 1.045 |
| PSQI_score | 1.096 | 1 | 1.047 |
| Age | 1.205 | 1 | 1.098 |
| Gender | 1.356 | 1 | 1.164 |
| Education level | 1.493 | 3 | 1.069 |
| Marital status | 1.265 | 2 | 1.061 |
| Whether retire | 1.009 | 1 | 1.005 |
| Career | 1.354 | 1 | 1.163 |
| Family income | 1.253 | 3 | 1.038 |
| Smoking status | 1.312 | 1 | 1.145 |
| Drinking status | 1.250 | 1 | 1.118 |
| Drink coffee | 1.061 | 1 | 1.030 |
| Taste | 1.098 | 1 | 1.048 |
| Calcium supplement | 1.085 | 1 | 1.042 |
| Entertainment | 1.322 | 3 | 1.048 |
| Social activity | 1.256 | 3 | 1.039 |
| Hypertension | 1.679 | 1 | 1.296 |
| Dyslipidemia | 1.309 | 1 | 1.144 |
| Heart disease | 1.242 | 1 | 1.114 |
| Stroke | 1.159 | 1 | 1.077 |
| Number of diseases | 2.662 | 2 | 1.227 |

**Supplementary Table 4.** Multiplicative interactions between B-complex vitamins intake and sleep quality on cognitive function stratified by 7 domains of PSQI

| **Variables** | **Model 1** |  | **Model 2** |  | **Model 3** |  |
| --- | --- | --- | --- | --- | --- | --- |
|  | OR [95% CI] | P value | OR [95% CI] | P value | OR [95% CI] | P value |
| **Subjective sleep quality** |  |  |  |  |  |  |
| VB index | 0.88 [0.63, 1.24] | 0.475 | 1.02 [0.73, 1.41] | 0.928 | 0.97 [0.70, 1.35] | 0.857 |
| Subjective sleep quality | 1.73 [1.41, 2.10] | <0.001 | 1.58 [1.29, 1.93] | <0.001 | 1.58 [1.29, 1.94] | <0.001 |
| Interaction effect | 0.64 [0.49, 0.83] | **0.001** | 0.66 [0.51, 0.85] | **0.001** | 0.67 [0.51, 0.86] | **0.002** |
| **Sleep latency** |  |  |  |  |  |  |
| VB index | 0.57 [0.47, 0.68] | <0.001 | 0.66 [0.55, 0.80] | <0.001 | 0.63 [0.52, 0.77] | <0.001 |
| Sleep latency | 2.12 [1.02, 1.24] | 0.020 | 1.21 [1.09, 1.34] | <0.001 | 1.20 [1.08, 1.33] | 0.001 |
| Interaction effect | 0.90 [0.78, 1.04] | 0.169 | 0.93 [0.82, 1.07] | 0.318 | 0.94 [0.82, 1.08] | 0.369 |
| **Sleep duration** |  |  |  |  |  |  |
| VB index | 0.48 [0.40, 0.58] | <0.001 | 0.56 [0.47, 0.67] | <0.001 | 0.54 [0.45, 0.65] | <0.001 |
| Sleep duration | 1.53 [1.38, 1.69] | <0.001 | 1.42 [1.28, 1.57] | <0.001 | 1.43 [1.29, 1.59] | <0.001 |
| Interaction effect | 1.07 [0.94, 1.21] | 0.309 | 1.08 [0.96, 1.22] | 0.186 | 1.09 [0.97, 1.23] | 0.145 |
| **Sleep efficiency** |  |  |  |  |  |  |
| VB index | 0.55 [0.47, 0.65] | <0.001 | 0.64 [0.55, 0.75] | <0.001 | 0.62 [0.53, 0.73] | <0.001 |
| Sleep efficiency | 1.76 [1.59, 1.95] | <0.001 | 1.72 [1.56, 1.91] | <0.001 | 1.72 [1.55, 1.91] | <0.001 |
| Interaction effect | 0.99 [0.87, 1.13] | 0.920 | 1.02 [0.90, 1.15] | 0.752 | 1.01 [0.89, 1.14] | 0.890 |
| **Sleep disturbances** |  |  |  |  |  |  |
| VB index | 0.38 [0.29, 0.48] | <0.001 | 0.43 [0.34, 0.55] | <0.001 | 0.42 [0.32, 0.53] | <0.001 |
| Sleep disturbances | 1.37 [1.14, 1.63] | 0.001 | 1.59 [1.31, 1.93] | <0.001 | 1.57 [1.29, 1.92] | <0.001 |
| Interaction effect | 1.39 [1.09, 1.76] | **0.007** | 1.44 [1.14, 1.79] | **0.002** | 1.45 [1.15, 1.81] | **0.002** |
| **Sleep medications** |  |  |  |  |  |  |
| VB index | 0.52 [0.45, 0.59] | <0.001 | 0.61 [0.53, 0.70] | <0.001 | 0.59 [0.52, 0.67] | <0.001 |
| Sleep medications | 1.26 [1.15, 1.37] | <0.001 | 1.20 [1.10, 1.31] | <0.001 | 1.20 [1.09, 1.30] | <0.001 |
| Interaction effect | 0.96 [0.85, 1.07] | 0.468 | 0.98 [0.87, 1.09] | 0.697 | 0.97 [0.87, 1.09] | 0.637 |
| **Daytime dysfunction** |  |  |  |  |  |  |
| VB index | 0.44 [0.37, 0.52] | <0.001 | 0.52 [0.44, 0.62] | <0.001 | 0.50 [0.42, 0.59] | <0.001 |
| Ddaytime dysfunction | 1.64 [1.46, 1.83] | <0.001 | 1.59 [1.42, 1.79] | <0.001 | 1.59 [1.42, 1.79] | <0.001 |
| Interaction effect | 1.19 [1.02, 1.37] | **0.021** | 1.21 [1.06, 1.39] | **0.006** | 1.22 [1.06, 1.40] | **0.004** |

**Supplementary Table 5.** Cognitive impairment risk by B-complex vitamins intake and sleep quality status.

| **Group** | **Model 1** | | **Model 2** | | **Model 3** | |
| --- | --- | --- | --- | --- | --- | --- |
|  | OR [95% CI] | P value | OR [95% CI] | P value | OR [95% CI] | P value |
| **High VB + Good Sleep Quality** | Reference |  | Reference |  | Reference |  |
| **Low VB + Good Sleep Quality** | 2.48 [1.90, 3.26] | <0.001 | 2.12 [1.61, 2.80] | <0.001 | 2.21 [1.67, 2.93] | <0.001 |
| **High VB + Poor Sleep Quality** | 2.09 [1.58, 2.79] | <0.001 | 2.16 [1.61, 2.90] | <0.001 | 2.17 [1.62, 2.92] | <0.001 |
| **Low VB + Poor Sleep Quality** | 4.60 [3.60, 5.94] | <0.001 | 4.22 [3.27, 5.51] | <0.001 | 4.35 [3.35, 5.70] | <0.001 |

**Supplementary Table 6.** Sensitivity analysis of cognitive impairment risk using alternative percentile thresholds for B-complex vitamins intake and sleep quality classification.

| **Percentile** | **Group** | **Model 1** | | **Model 2** | | **Model 3** | |
| --- | --- | --- | --- | --- | --- | --- | --- |
|  |  | **OR [95% CI]** | **P value** | **OR [95% CI]** | **P value** | **OR [95% CI]** | **P value** |
| **25/75th** | **High VB + Good Sleep Quality** | Reference |  | Reference |  | Reference |  |
|  | **Low VB + Good Sleep Quality** | 2.28 [1.88, 2.77] | <0.001 | 1.73 [1.41, 2.12] | <0.001 | 1.81 [1.47, 2.22] | 0.001 |
|  | **High VB + Poor Sleep Quality** | 1.96 [1.56, 2.45] | <0.001 | 1.90 [1.50, 2.40] | <0.001 | 1.93 [1.52, 2.43] | <0.001 |
|  | **Low VB + Poor Sleep Quality** | 7.10 [5.44, 9.22] | <0.001 | 4.62 [3.47, 6.12] | <0.001 | 4.78 [3.56, 6.37] | <0.001 |
| **33.3/66.7th** | **High VB + Good Sleep Quality** | Reference |  | Reference |  | Reference |  |
|  | **Low VB + Good Sleep Quality** | 2.46 [2.01, 3.02] | <0.001 | 1.95 [1.58, 2.41] | <0.001 | 2.02 [1.63, 2.51] | <0.001 |
|  | **High VB + Poor Sleep Quality** | 1.96 [1.55, 2.48] | <0.001 | 2.02 [1.58, 2.57] | <0.001 | 2.04 [1.60, 2.61] | <0.001 |
|  | **Low VB + Poor Sleep Quality** | 5.93 [4.70, 7.49] | <0.001 | 4.48 [3.49, 5.74] | <0.001 | 4.55 [3.53, 5.86] | <0.001 |
| **75/25th** | **High VB + Good Sleep Quality** | Reference |  | Reference |  | Reference |  |
|  | **Low VB + Good Sleep Quality** | 3.56 [2.28, 5.89] | <0.001 | 3.39 [2.12, 5.71] | <0.001 | 3.44 [2.16, 5.81] | <0.001 |
|  | **High VB + Poor Sleep Quality** | 2.90 [1.77, 4.97] | <0.001 | 3.14 [1.87, 5.50] | <0.001 | 3.02 [1.80, 5.29] | <0.001 |
|  | **Low VB + Poor Sleep Quality** | 6.23 [4.07, 10.13] | <0.001 | 6.28 [4.02, 10.43] | <0.001 | 6.34 [4.05, 10.54] | <0.001 |

**Supplementary Table 7.** Absolute risk of cognitive impairment by B-complex vitamins intake and sleep quality status.

| **Group** | **Cases** | **Total** | **Model 1** | **Model 2** | **Model 3** |
| --- | --- | --- | --- | --- | --- |
|  | N | N | AR (%) [95% CI] | AR (%) [95% CI] | AR (%) [95% CI] |
| **High VB + Good Sleep Quality** | 83 | 2,522 | 3.29 [2.66, 4.06] | 2.71 [2.16, 3.38] | 2.59 [2.07, 3.24] |
| **Low VB + Good Sleep Quality** | 172 | 2,209 | 7.79 [6.74, 8.98] | 5.56 [4.73, 6.54] | 5.55 [4.70, 6.53] |
| **High VB + Poor Sleep Quality** | 125 | 1,881 | 6.65 [5.60, 7.86] | 5.67 [4.73, 6.79] | 5.45 [4.53, 6.54] |
| **Low VB + Poor Sleep Quality** | 297 | 2,194 | 13.54 [12.17, 15.03] | 10.52 [9.28, 11.91] | 10.37 [9.11, 11.77] |
